# Nmnat3 Is Dispensable in Mitochondrial NAD Level Maintenance *In Vivo*

**DOI:** 10.1371/journal.pone.0147037

**Published:** 2016-01-12

**Authors:** Masashi Yamamoto, Keisuke Hikosaka, Arshad Mahmood, Kazuyuki Tobe, Hideo Shojaku, Hidenori Inohara, Takashi Nakagawa

**Affiliations:** 1 Frontier Research Core for Life Sciences, University of Toyama, Toyama 930–0194, Japan; 2 Department of Otorhinolaryngology-Head and Neck Surgery, Osaka University Graduate School of Medicine, Osaka 565–0871, Japan; 3 First Department of Internal Medicine, Graduate School of Medicine and Pharmaceutical Science for Research, University of Toyama, Toyama 930–0194, Japan; 4 Department of Otorhinolaryngology, Head and Neck Surgery, Graduate School of Medicine and Pharmaceutical Science for Research, University of Toyama, Toyama 930–0194, Japan; Rutgers New Jersey Medical School, UNITED STATES

## Abstract

Nicotinamide adenine dinucleotide (NAD) is an essential co-enzyme mediating various enzymatic reactions. Mitochondrial NAD particularly occupies a considerable amount of total NAD in cells, and serves as a co-enzyme in tricarboxylic acid cycle (TCA cycle), β-oxidation, and oxidative phosphorylation. Despite the importance of mitochondrial NAD, its synthesis pathway remains unknown. It has been proposed that NAD synthesis enzyme, Nmnat3, was localized in mitochondria, but its physiological relevance to the metabolism in mitochondria was not fully elucidated. Previously, we have reported that murine Nmnat3 protein was strongly expressed in the cytoplasm of mature erythrocytes, in which mitochondria were absent, and Nmnat3-deficient mice (Nmnat3-KO mice) exhibited splenomegaly and hemolytic anemia due to reduced NAD levels in mature erythrocytes. These results challenged the role of Nmnat3 in mitochondrial NAD synthesis. In this study, we demonstrated that mitochondrial NAD levels in various tissues, except for red blood cells, were unchanged in Nmnat3-KO mice. We also analyzed the metabolites in glycolysis and TCA cycle and found that there were no differences between Nmnat3-KO and WT mice. In addition, the aged Nmnat3-KO mice had comparable NAD levels to that observed in WT mice. Our results indicated that Nmnat3 is dispensable in the maintenance of mitochondrial NAD levels, and that other NAD regulatory pathways may exist in mitochondria.

## Introduction

Mitochondria are energy centers producing ATP through oxidative phosphorylation [1]. In mammalian cells, NAD is reduced to a form of NADH by TCA cycle or β-oxidation in mitochondria. Then, NADH is oxidized through the electron transport chain generating ATP [2,3]. It has been considered that 40%–70% of NAD in cells resides in the mitochondria [4–6]. However, the mammalian mitochondrion is an organelle, which has a lipid bilayer membrane, and the inner membrane is impermeable to pyridine nucleotides including NAD [7–9]. Therefore, it has been considered that NAD is likely to be synthesized inside mitochondria [10]. Even though numerous studies have tried to identify NAD synthesis activities in mitochondria, it is arguable whether mitochondria have the NAD synthesis enzymes or not [9–15].

In organisms, NAD can be synthesized through *de novo* and salvage pathways. In salvage pathway, Nampt (Nicotinamide phosphoribosyl- transferase) generates nicotinamide mononucleotide (NMN) by transferring a phosphoribosyl moiety from phosphoribosyl pyrophosphate (PRPP) to nicotinamide (NAM), and then nicotinamide mononucleotide adenylyltransferase (Nmnat) generates NAD from NMN and ATP [16]. In mammalian cells, there are three Nmnat isozymes (Nmnat1-3), which are encoded by different nuclear genes [17–20]. Previous studies have demonstrated that human Nmnat isozymes have different subcellular localizations. While Nmnat1 and Nmnat2 reside in the nucleus and cytoplasm (including golgi), where earlier biochemical studies have identified considerable amounts of Nmnat activities, Nmnat3 is located in the mitochondria [20,21]. However, these data were collected using the models of Nmnat3 overexpressing cultured cell. Thus, the localization of endogenous Nmnat3 in cells and tissues was undetermined.

Previously, we have demonstrated that Nmnat3 was strongly expressed in mature erythrocytes, which lacked mitochondria, and Nmnat3-deficient mice exhibited splenomegaly and hemolytic anemia [22]. These results triggered the question whether Nmnat3 is indeed responsible for NAD metabolism in mitochondria or not. In this study, we examined the role of Nmnat3 in mitochondria using Nmnat3-deficient mice. We found that Nmnat3 was mainly localized in the cytoplasm and was not essential for the maintenance of mitochondrial NAD homeostasis. These results further question the origin of mitochondrial NAD.

## Material and Methods

### Animal experiments

Nmnat3-defieinct (Nmnat3 KO) mice were described previously [22]. Mice were maintained under controlled temperature and standard light conditions (12h:12h light-dark cycle) and were allowed free access to water and food. All animal experiments were approved by the Animal Experiment Committee at University of Toyama and were carried out in accordance with the Guidelines for the Care and Use of Laboratory Animals at University of Toyama, which were based on international policies.

### Isolation of mitochondria

Isolation of mitochondria from mouse tissues was described elsewhere [23,24]. In brief, whole liver was excised from WT and Nmnat3 KO mice, and then homogenized in Buffer LA (0.3M Mannitol, 10mM HEPES pH7.4 and 0.2mM EDTA pH8.0). Homogenates were centrifuged at 750*g* for 10min at 4˚C, and the supernatant were centrifuged again at 7,000*g* for 10 min at 4˚C. These centrifugations were repeated twice. The pellets were dissolved in Buffer LB (0.3M Mannitol and 10mM HEPES pH7.4), and the concentration was measured by Qubit Fluorometer (Life Techcnolgies). Skeletal muscles were excised from hind limb, and were homogenized in Buffer MA (67mM Sucrose, 50mM Tris-HCl pH7.4, 50mM KCl and 10mM EDTA), followed by the centrifugation by the same scheme described above. The pellet was dissolved in Buffer MB (250mM Sucrose, 10mM Tris-HCl pH7.4 and 3mM EGTA) and used as mitochondria. The supernatant after the first centrifugation was centrifuged again at 18,000*g* for 10 min at 4˚C and used as cytoplasmic fraction for Western blotting experiments.

### Western blotting experiment

Whole tissue lysates were prepared from WT mice. Before harvesting tissues, mice were systemically perfused with D-PBS(-) (Nacalai) via inferior vena cava (IVC) to eliminate the contamination of blood. Harvested whole tissues were grinded by Multi Beads Shocker (Yasui Kikai) with RIPA buffer (150 mM NaCl, 1.0% NP-40, 1mM EDTA, 0.1% SDS, 0.1% sodium deoxycholate, and 50 mM Tris-HCl, pH 8.0), and were subjected to Western blotting. Antibodies used for Western blotting experiments included anti-GAPDH (Sigma), anti-CoxIV (Cell Signaling) and anti-Grp75 (Abcam). Anti-mouse Nmnat3 rat monoclonal antibody (clone R88) was described previously [22]. HRP-conjugated secondary antibodies were obtained from Millipore. PVDF membrane (Millipore) was used for blotting and signals were detected by LAS4000 mini digital imager (GE Health Care).

### Metabolites extraction for LC/MS and GC/MS

Whole tissues were grinded by Multi Beads Shocker (Yasui Kikai) with LC-MS grade methanol and water in the proportion of 1:1 (by volume). After the centrifugation, the supernatant was mixed with the same volume of chloroform, and the aqueous phase was dried by SpeedVac SPD1010 (Thermo). For the LC/MS analysis, the dried sample was reconstituted by 50 μl LC/MS grade water (Wako) and filtered with 0.45 μm Millex filter unit (Millipore). For the GC/MS analysis, two-step derivatization was carried out. First, carbonyl functional groups were protected by methoximation using 20 μL of 20 mg/mL solution of methoxyamine hydrochloride in pyridine at 30°C for 90 min. Next, the samples were derivatized using 80 μL of N-methyl-N-trimethylsilyltrifluoroacetamide with 1% trimethylchlorosilane (MSTFA + 1% TMCS, Pierce) at 37°C for 30 min.

### Metabolites measurement by LC/MS and GC/MS

Polar metabolites, including NAD, NADH, GSH, GSSG, ATP and AMP, were determined by multiple reaction monitoring (MRM) mode using Agilent 6460 Triple Quad mass spectrometer coupled to Agilent 1290 HPLC system. Chromatographic conditions were used as previously described [22]. Pyruvate, lactate and TCA cycle intermediates were assayed by selected ion monitoring (SIM) mode using Agilent 5977 MSD Single Quad mass spectrometer coupled to Agilent 7890 Gas chromatography. As a GC condition, a 30 m long DB5-MS column with 10 m Duragard precolumn was used with 0.25 mm diameter and 0.25 μm film thickness. A constant flow rate of 1.1 mL/min helium was used as carrier gas. The temperature program started at 60°C for 1 min, increased at 10°C/min to 325°C, and held at 325°C for 10 min. The injector and detector temperatures were set at 290°C. Semi-quantification of metabolites level was calculated by integrated sum of area using MassHunter Quantitative software (Agilent).

### Enzymatic activity assay

The proteins extracted from whole tissues or mitochondria were used as samples. These proteins were dialyzed against dialysis buffer (25mM Tris-Cl pH 7.4, 100mM NaCl and 10% glycerol) to remove interfering endogenous metal ions and metabolites. The condition of assay was determined by previous report [25]. Briefly, the total 100μl of reaction mixture contains 10μl of dialyzed sample, 1mM ATP and 1mM NMN in final concentration. Metal ions were used at 0.05mM for MgCl_2_ in final concentration. The reaction was terminated by adding 200μl 0.5N perchloric acid (PCA) at 0 and 30 min. After centrifugation, supernatant was collected and the formed NAD amount was measured by LC-MS/MS method described above. Total pool size of Nmnat activities contained in whole cell and mitochondria was calculated by multiplying the unit activity by wet weight of total tissue or mitochondria.　

### Real-time quantitative PCR

Total RNAs were extracted from mouse tissues using TRI Reagent (Molecular Research Center, Inc.). cDNA was prepared using ReverTra Ace qPCR RT Master Mix with gDNA Remover (Toyobo, Japan) according to the supplier’s protocol. Real-time PCR was carried out using THUNDERBIRD SYBR qPCR Mix (Toyobo) on Thermal Cycler Dice Real Time System II (Takara Bio). Quantification was done by Delta Delta Ct method, and *Rpl13a* gene was used as a reference gene. Primers used in qPCR are listed in Table 1.

**Table 1:** Primers for real time qPCR.

**Nmnat1**	FWD: GTGGAGACTGTGAAGGTGCTC
	REV: GTGAGCTTTGTGGGTAACTGC
**Nmnat2**	FWD: TGGAGCGCTTCACTTTTGTA
	REV: CGATCTCCTCATACCGCATC
**Nmnat3**	FWD: CACCAAACAGGAAGGTACCA
	REV: AAGCCACCAGGTCTTTCTTC
**Rpl13a (ribosomal protein L13a)**	FWD: AGCGCCTCAAGGTGTTGGA
	REV: GAGTGGCTGTCACTGCCTGGTA

The primers used in real time qPCR for Nmnat1, Nmnat2 and Nmnat3. RPL13a was used as a reference gene.

### Histological staining

After the excision, the tissues were fixed with 4% paraformaldehyde (Wako), and embedded in paraffin. The paraffin sections of 3 μm thickness were subjected to hematoxylin and eosin staining. Sample slides were observed using BX61 microscope (Olympus, Japan).

### Statistic Analysis

Analysis was performed using an unpaired or paired Student’s t-test. Data are expressed as the mean ± SD, and significant differences are confirmed statistically when *p*-value is less than 0.05.

## Results

### Nmnat3 is not responsible for the maintenance of mitochondrial NAD levels

**Fig 1:**
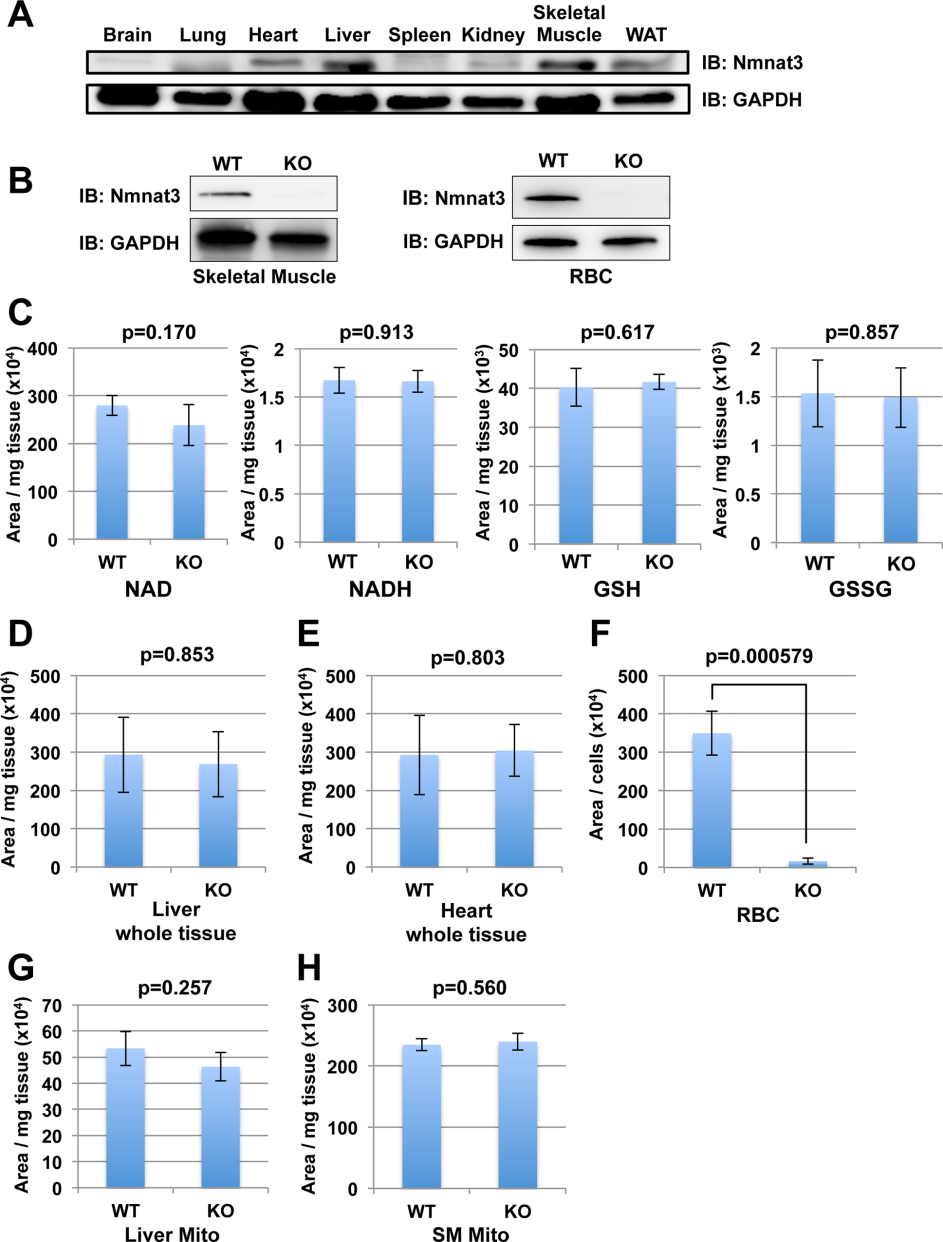
Deficiency of Nmnat3 in mice does not decrease the NAD level in mitochondria. (A) Immunoblot analysis of Nmnat3 expression in indicated tissues from wild-type (WT) mouse. GAPDH was used as loading control. (B) Immunoblot analysis of Nmnat3 in skeletal muscle (Left) and RBC (Right) prepared from WT and Nmnat3 KO mice. GAPDH was used as a loading control. (C) Metabolites, including NAD, NADH GSH and GSSG, were measured by LC/MS using skeletal muscle samples prepared from 5-month-old WT and Nmnat3 KO mice. Data are presented as mean ± SD (n = 4 for each group). (D-F) Semi-quantification of NAD levels in liver (D), heart (E) and red blood cell (F) prepared from 5-month-old WT and Nmnat3 KO mice. Data are presented as mean ± SD (n = 4 for each group). (G and H) Semi-quantification of mitochondrial NAD levels in liver (G) and skeletal muscle (H) prepared from 5-month-old WT and Nmnat3 KO mice. Data are presented as mean ± SD (n = 4 for each group).

Although several reports suggested that Nmnat3 was the mitochondrial isoform of Nmnat in cultured cells, the physiological function of Nmnat3 in mitochondria, especially *in vivo*, remains unclear. To investigate the role of Nmnat3 in mitochondrial NAD synthesis, we examined the tissue distribution of Nmnat3 in wild-type (WT) mice by Western blotting with anti-mouse Nmnat3 monoclonal antibody. To avoid the contamination of erythrocyte where Nmnat3 was abundant, we employed *in vivo* systemic perfusion with PBS before harvesting tissues from mice. As reported earlier [22], the expression level of Nmnat3 protein varied between tissues (Fig 1A). Skeletal muscles, heart, kidney, and liver showed certain amount of expression of Nmnat3 (Fig 1A). Notably, some tissues including brain and spleen had negligible Nmnat3 expression.

It is well known that Nmnat is an important enzyme that is responsible for both *de novo* and salvage pathway of NAD synthesis [26]. To investigate whether Nmnat3 contributes to mitochondrial NAD metabolism *in vivo*, we employed whole body Nmnat3-deficient mice (Nmnat3-KO mice). We confirmed the deletion of Nmnat3 protein in Nmnat3-KO mice by western blotting using anti-Nmnat3 antibody (Fig 1B). At first, we examined the total NAD level in liver, skeletal muscle, heart, and red blood cells where Nmnat3 proteins were expressed. As previously reported [22], NAD level in red blood cells from Nmnat3-KO mice was notably lower than that from WT mice (Fig 1F). However, there were no significant differences in NAD levels between Nmnat3-KO and WT mice in other tissues such as liver, skeletal muscle, and heart (Fig 1C–1E). We also examined the level of NADH, GSH (reduced form of glutathione) and GSSG (oxidized form of glutathione) in skeletal muscle, but there were no significant differences between WT and Nmnat3-KO mice. These results indicated that the redox state was not altered in Nmnat3-KO mice (Fig 1C).

Next, we examined mitochondrial NAD levels in Nmnat3-KO mice. We isolated intact whole mitochondria from liver and skeletal muscle of both Nmnat3-WT and KO mice, and measured the NAD levels by LC/MS. However, we found no significant differences in mitochondrial NAD levels (Fig 1G and 1H). These results indicated that Nmnat3 did not contribute to the maintenance of NAD levels in most tissues except red blood cells.

### Deficiency of Nmnat3 has no impact on glycolysis and TCA cycle in skeletal muscle

**Fig 2:**
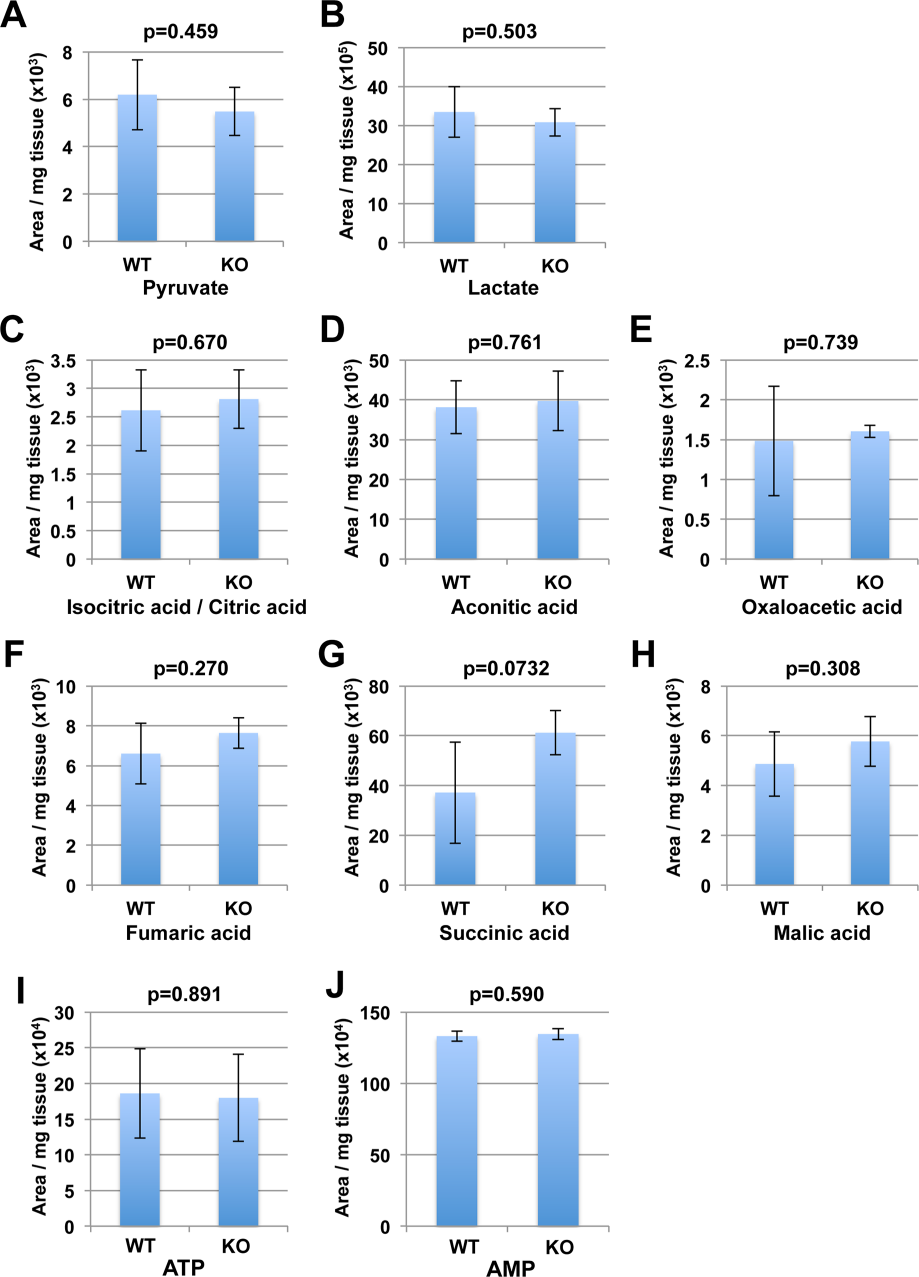
The glycolysis pathway and TCA cycle were normally maintained in Nmnat3 KO mice. Metabolites in glycolysis pathway (A and B) and TCA cycle (C-H) were measured by SIM mode operated-GC/MS using skeletal muscle samples from 5-month-old WT and Nmnat3 KO mice (n = 4 for each group). Metabolites measured by GC/MS included pyruvate (A), lactate (B), isocitric acid/ citric acid (C), aconitic acid (D), oxaloacetic acid (E), fumaric acid (F), succinic acid (G), and malic acid (H). Data are presented as mean ± SD (n = 4 for each group). (I and J) Levels of ATP (I) and AMP (J) in skeletal muscle were measured by LC/MS. Data are presented as mean ± SD (n = 4 for each group).

Previously, we demonstrated that the glycolysis pathway was blocked in red blood cells from Nmnat3-KO mice because NAD serves as the co-enzyme for glyceraldehyde phosphate dehydrogenase (GAPDH) and lactate dehydrogenase (LDH) in the glycolysis pathway [22]. In addition, NAD is also used as a co-enzyme in TCA cycle [27]. Therefore, we investigated the influence of Nmnat3 deficiency on glycolysis and TCA cycle in skeletal muscle. As we measured the metabolites of glycolysis using GC/MS, we observed no significant differences in the levels of pyruvate and lactate, which were involved in the reaction mediated by LDH (Fig 2A and 2B). In TCA cycle, the level of intermediate metabolites including isocitric acid/ citric acid, aconitic acid, oxalaoacetic acid, fumaric acid, succinic acid, and malic acid were unchanged in Nmnat3-KO mice compared to those observed in WT mice (Fig 2C–2H). These results demonstrated that the glycolysis pathway and TCA cycle, in which NAD was required as a co-enzyme, were not altered in Nmnat3-KO mice. In line with these results, ATP and AMP levels of skeletal muscle in Nmnat3-KO mice were comparable with that in WT mice (Fig 2I and 2J). Based on these data, we concluded that Nmnat3 deficiency had no impact on glycolysis pathway and TCA cycle in skeletal muscle.

### Nmnat3 partially contributes to Nmnat activity in mitochondria

**Fig 3:**
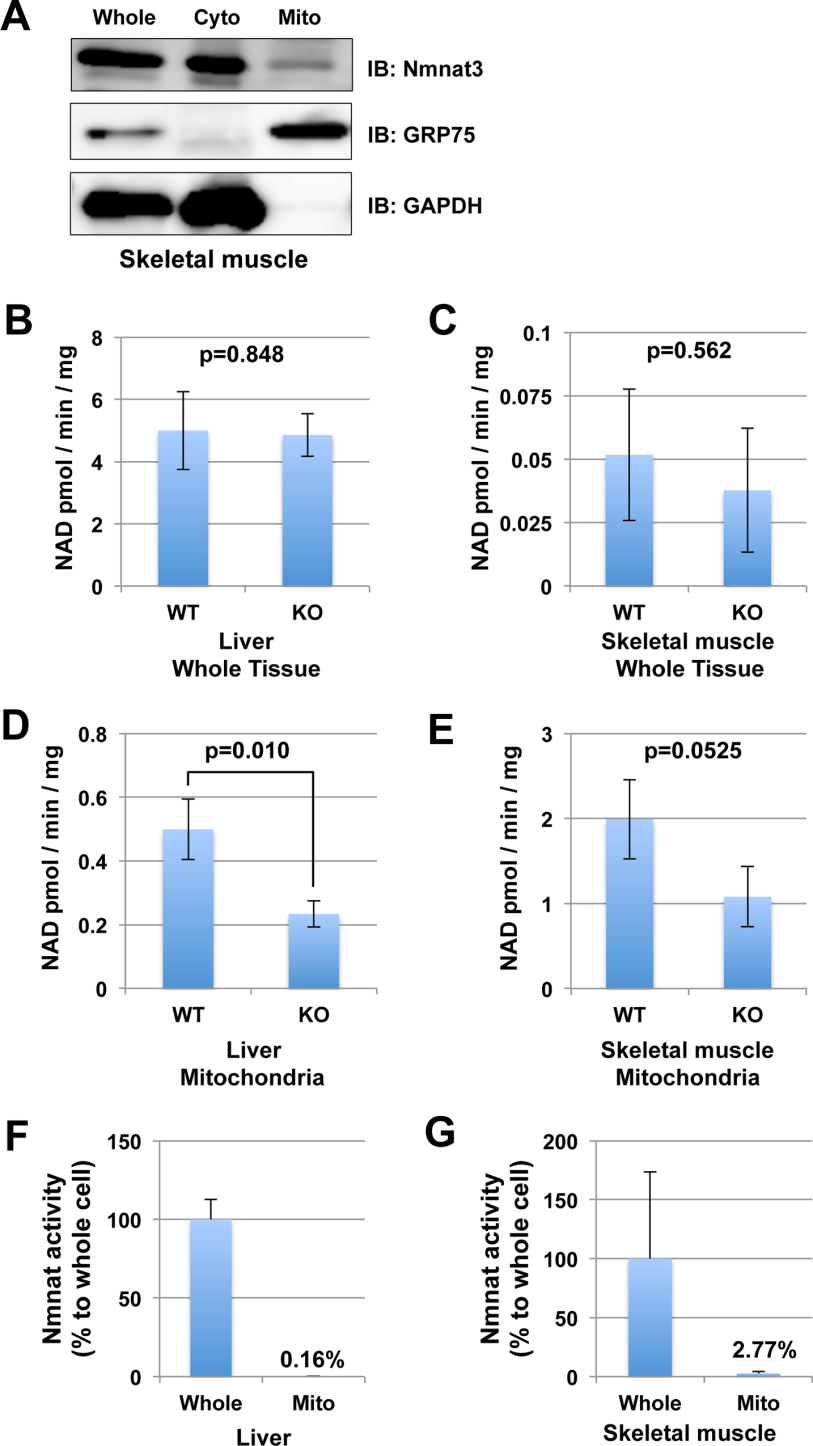
Nmnat3 partially contributes to the Nmnat activity in mitochondria. (A) Subcellular fractionation of endogenous Nmnat3 protein in skeletal muscle. GAPDH was used as a cytoplasmic fraction marker, and GRP75 (mitochondrial HSP70) was used as a mitochondrial fraction marker. (B and C) Nmnat activities of total tissue lysates from liver (B) and skeletal muscle (C) were measured by enzymatic activity assay. For this assay, tissue lysates were prepared from WT and Nmnat3 KO mice. Samples were dialyzed to remove endogenous metal ion and metabolites. Data are presented as mean ± SD (n = 4 for each group). (D and E) Mitochondrial Nmnat activities from liver (B) and skeletal muscle (C) were also measured in the same way as above. (F and G) Ratio of Nmnat activity in mitochondria against that in whole cell from WT liver (F) and WT skeletal muscle. Data are presented as mean ± SEM (n = 3 for each group).

We found that Nmnat3 was not responsible for the maintenance of mitochondrial NAD levels. Accordingly, we investigated whether Nmnat3 was actually a mitochondrial Nmnat isozyme or not. To address this question, we performed subcellular fractionation using skeletal muscle samples from WT mice. We found that the majority of Nmnat3 protein was present in the cytoplasmic fraction but also sparsely in the mitochondrial fraction (Fig 3A). Next, we probed whether NAD synthesis activity in mitochondria correlated with Nmnat3. To assess this, we measured the enzymatic activity of Nmnat in liver and skeletal muscle. At first, we used whole tissue lysate for enzymatic activity assay, but there were no significant differences between Nmnat3-KO and WT mice in both liver and skeletal muscle (Fig 3B and 3C). On the other hand, Nmnat activity in liver mitochondria from Nmnat3-KO mice was only half of that observed in WT mice (Fig 3D). Nmnat activity in skeletal muscle mitochondria from Nmnat3-KO mice also showed a similar trend, but it was not statistically significant (Fig 3E). We also measured the ratio of mitochondrial Nmnat activity against whole cell in liver and skeletal muscle from WT mice. Consistent with previous report [11], the pool size of mitochondrial Nmnat activity was very minor compared with that in whole cell (Fig 3F and 3G). The calculated ratio of Nmnat activity in mitochondria to that in whole cell were 0.16% in liver (Fig 3F) and 2.77% in skeletal muscle (Fig 3G). These results suggested that Nmnat3 partially occupied the Nmnat activity in mitochondria, but the contribution of intra-mitochondrial Nmnat acitivity to mitochondrial NAD homeostasis was limited.

### Nmnat1 and Nmnat2 are not up regulated in Nmnat3-KO mice at mRNA level

**Fig 4:**
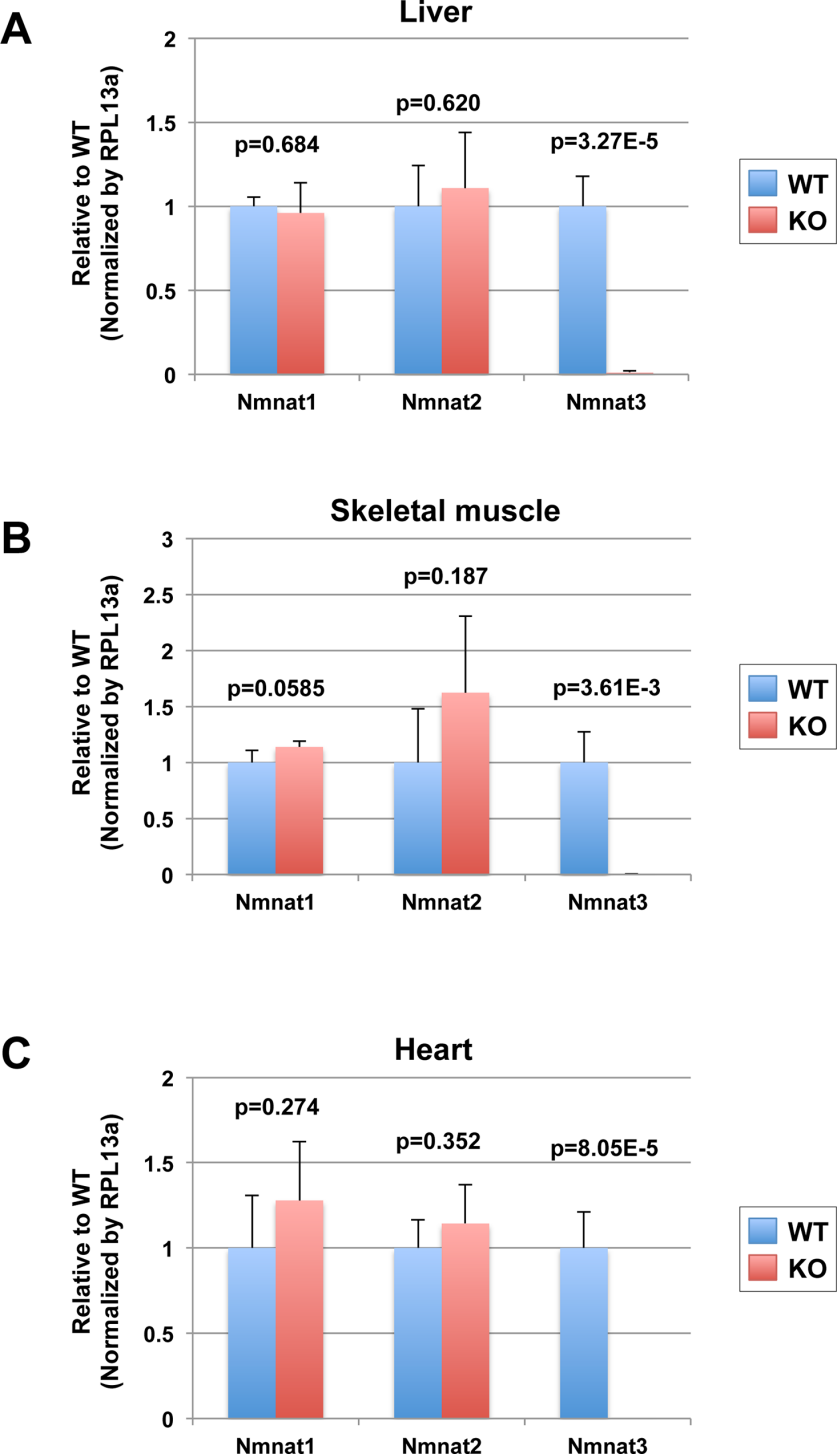
Nmnat1 and Nmnat2 are not up-regulated in Nmnat3 KO mice. (A-C) Real-time quantitative PCR analysis for mRNA level of Nmnat1, Nmnat2 and Nmnat3. Total RNA was isolated from liver (A), skeletal muscle (B) and heart (C) of WT and Nmnat3 KO mice. *Rpl13a* gene was used as a reference gene, and data are presented as a relative value to WT for each gene (n = 4 for each group).

To investigate whether Nmnat1 and/or Nmnat2 were up regulated in Nmnat3-KO mice to compensate the absence of Nmnat3, we checked the mRNA levels of Nmnat1, Nmnat2 and Nmnat3 in tissues from Nmnat3 WT and KO mice by real-time qPCR. As expected, the mRNA of Nmnat3 was absent in liver, skeletal muscle and heart from Nmnat3-KO mice (Fig 4A–4C). However, we found that mRNA levels of both Nmnat1 and Nmnat2 were not significantly changed between Nmnat3 WT and KO mice (Fig 4A–4C). These results indicated that the contribution of Nmnat3 in NAD homeostasis was relatively minor compared to other Nmnat isoforms.

### NAD levels are not changed even in aged Nmnat3-KO mice

**Fig 5:**
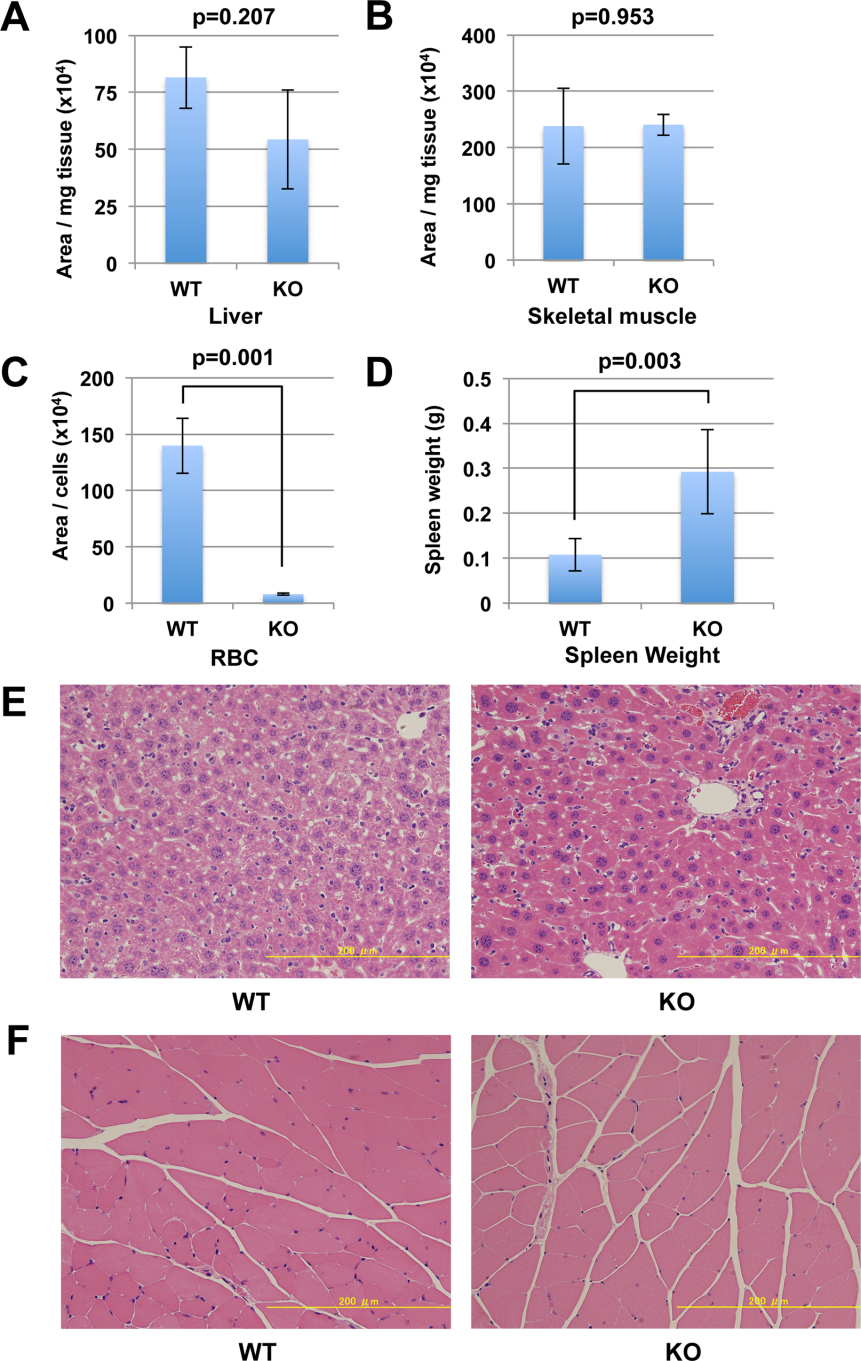
NAD level is not changed even in the aged Nmnat3 KO mice. (A-C) Semi-quantification of NAD levels in liver (A), skeletal muscle (B) and red blood cells (C) from 21-month-old WT and Nmnat3 KO mice. Data are presented as mean ± SD (n = 4 for each group). (D) Spleen weight was calculated using 21-month-old WT and Nmnat3 KO mice (n = 4 for each group). (D and E) Hematoxylin-Eosin (HE) staining of liver (D) and skeletal muscle (E) sections prepared from 21-month-old WT and Nmnat3 KO mice. Scale bar represents 200μm.

Finally, to examine the long-term effect of Nmnat3 deficiency on NAD metabolism, we analyzed the NAD levels in aged Nmnat3-KO and WT mice (21 months old). Nmnat3-KO mice could survive for even more than 2 years, and there were no recognizable differences in appearance between Nmnat3-KO and WT mice (data not shown). We examined NAD levels in whole liver and skeletal muscle, but Nmnat3-KO mice had comparable NAD levels to WT mice (Fig 5A and 5B). As with young mice, NAD levels in red blood cells from the aged Nmnat3-KO mice remained significantly lower than those from WT mice (Fig 5C). Next, to investigate the physiological changes in the tissues of the aged Nmnat3-KO mice, we performed histological analysis. The aged Nmnat3-KO mice exhibited splenomegaly as we reported earlier in young Nmnat3-KO mice (Fig 5D). However, histological examinations in liver (Fig 5E) and skeletal muscle (Fig 5F) could not detect any apparent differences between Nmnat3-KO and WT mice. These results implied that NAD metabolism was not altered even in the aged Nmnat3-KO mice.

## Discussion

Although mitochondrial NAD homeostasis is extremely important for energy production, the origin of mitochondrial NAD remains unclear. In mammalian cells, NAD is synthesized thorough *de novo* and salvage pathways, and Nmnats are involved in both the pathways [28]. Classical biochemical studies identified the majority of Nmnat activity to be in the nucleus and cytoplasm, but scarce in mitochondria [11]. Therefore, whether mitochondria actually retain Nmnat activity or not is debatable. Some studies confirmed Nmnat activity in mitochondria using a biochemical enzymatic assay [9,10]. However, it was difficult to exclude the possibility of contamination from other cellular compartments, particularly the nucleus. Moreover, the exchange reactions by glycohydrolase, which was potentially contaminated from microsomes, also resulted in formation of NAD rather than synthesis [13]. Thus, biochemical analyses had certain limitations in ascertaining the origin of mitochondria Nmnat activity. Although the molecular identity of mitochondrial Nmnat had not been established for a long time, in 2004, Zhang et. al. reported a novel human isoform of Nmnat, PNAT3 (alternative name of Nmnat3) [20]. They characterized PNAT3 and found that overexpressed PNAT3 was localized in mitochondria in Hela cells. Other study groups also confirmed similar results [21]. However, most of the evidences in these studies were obtained using transiently transfected cultured cells. Interestingly, a recent paper demonstrated that Nmnat3 knockdown by siRNA in cultured cells had no effect on either total or mitochondrial Nmnat activity [29]. On the other hand, the knockdown of Nmnat1 significantly decreased the total and nucleus Nmnat activity, but not mitochondrial activity [29]. These results also questioned the crucial role of Nmnat3 in mitochondrial NAD maintenance. Therefore, information regarding endogenous Nmnat3 *in vivo* was required for further validation.

Previously, we have reported that murine Nmnat3 protein was strongly expressed in the cytoplasm of mature erythrocytes, in which mitochondria are absent [22]. This result was consistent with the previous study reporting that human Nmnat3 was dominant in erythrocytes [30]. In the present study, we demonstrated that mitochondrial NAD levels in various tissues, except for red blood cells, were unchanged between Nmnat3-KO and WT mice. In addition, we also analyzed the metabolites of the glycolysis pathway and TCA cycle, but we found that there were no differences between Nmnat3-KO and WT mice. These results suggested that Nmnat3 was dispensable in the maintenance of mitochondrial NAD level. We also examined the Nmnat activity in mitochondria. Surprisingly, mitochondria actually retained the Nmnat activities and these activities were decreased in Nmnat3-KO mitochondria. One of the possibilities of how the mitochondrial NAD level was maintained in Nmnat3-KO mice is that other Nmnat isozymes, such as Nmnat1 and/or Nmnat2, are imported to the mitochondria and generate NAD in the mitochondria matrix. In general, mitochondrial matrix proteins have targeting sequences in their N-terminus [31]. While Nmnat3 retains the probable mitochondrial targeting sequence, Nmnat1 and Nmnat2 have no apparent targeting sequences when they are investigated using MITOPROT II program (https://ihg.gsf.de/ihg/mitoprot.html) [32]. Currently, there is no evidence that Nmnat1 and Nmnat2 are localized in the mitochondria. We also revealed that mRNA levels of both Nmnat1 and Nmnat2 were comparable between Nmnat3 WT and KO mice (Fig 4A–4C). Nevertheless, it is important to determine the protein level of Nmnat1 and Nmnat2 in Nmnat3 KO mice in future. In addition, the contributions of Nmnat1 and Nmnat2 in mitochondrial NAD homeostasis *in vivo* should be investigated using Nmnat1 or Nmna2 tissue specific conditional knockout mice for future. Recently, another member of the Nmnat protein family, Pof1, was found in *Saccharomyces cerevisiae* [33]. Although the mammalian homologue of Pof1 has not been identified, we cannot exclude the existence of new putative mitochondrial Nmnats at this moment.

The other explanation of how the mitochondrial NAD level was maintained in the absence of Nmnat3 is the direct incorporation of NAD into the mitochondria. Indeed, our results also indicated that the contribution of intra-mitochondrial Nmnat activity to mitochondrial NAD homeostasis was limited. Mitochondrial NAD occupies around 50% of total NAD in whole cell. It is doubtful whether this marginal Nmnat activity in mitochondria is enough to maintain the huge mitochondrial NAD pool. Thus, our results would rather support the idea that mitochondrial NAD is synthesized outside, and incorporated into mitochondria. Currently, it is generally accepted that NAD cannot cross the inner membrane of mitochondria [7–9]. However, under certain conditions, the uptake of NAD into mitochondria was observed in mammals [34–36]. For instance, the incorporation of NAD was dependent on the energy-state of mitochondria [36]. When isolated mitochondria were incubated with respiration substrates, NAD was incorporated. On the other hand, antimycin A, an inhibitor of the mitochondrial electron transport chain, abolished the incorporation of NAD [36]. Another study demonstrated that Ca^2+^ also stimulates the incorporation of NAD in an energy-state independent manner [35]. Bernadi had reported that the mitochondrial permeability pore (mPTP) opening by Ca^2+^ stimulation significantly decreased the NAD content in isolated mitochondria. This process was completely inhibited by nicotinamide, which is an inhibitor of NAD glycohydrolase [37]. The mPTP is a type of putative mitochondrial inner membrane pore, which allows the permeability of all small molecules of molecular weight <1500 Da. [38]. Therefore, mPTP possibly functions as a NAD transporter under certain circumstances and regulates NAD metabolism in mitochondria.

Although these studies suggested the existence of a NAD incorporation system at the inner membrane of the mitochondria, the exact molecular identity of a NAD transporter has not yet been found in mammals. In lower organisms, such as yeast and plants, mitochondria NAD transporters have been reported [39,40]. However, the specificity of these transporters for NAD was not high enough, and the exact role *in vivo* is unclear. Besides, the mammalian homologues of these transporters have not yet been found. Thus, the existence of a mammalian NAD transporter is still elusive, and further studies are necessary.

In conclusion, Nmnat3 deficiency in mice did not change the NAD levels in mitochondria. In addition, the glycolysis pathway and TCA cycle were also normally retained in Nmnat3-KO mice. It was also reported that knockdown of human Nmnat3 in cultured cells did not reduce the NAD levels in mitochondria [29]. However, we do not exclude the possibility that Nmnat3 may act as a backup for mitochondrial NAD homeostasis under certain stress conditions such as oxidative stress or genotoxic stress, and further studies are required to uncover this issue. Thus, collectively, it is implied that Nmnat3 is not essentially involved in mitochondrial NAD synthesis under the physiological condition. The regulatory pathway of the mitochondrial NAD remains unknown. In the future, we intend to reveal the molecular identity of mitochondrial NAD synthesis enzymes and/or a mitochondrial NAD transporter.
